# Bringing new medicines to women with epithelial ovarian cancer: what is the unmet medical need?

**DOI:** 10.1186/s40661-017-0050-0

**Published:** 2017-09-07

**Authors:** Thomas J. Herzog, Bradley J. Monk

**Affiliations:** 1 University of Cincinnati Cancer Institute, University of Cincinnati, Medical Sciences Bldg, Suite 2005H, ML0662, 231 Albert Sabin Way, Cincinnati, OH 45267-0662 USA; 2 Arizona Oncology (US Oncology Network), University of Arizona College of Medicine and Creighton University School of Medicine at Dignity Health St. Joseph’s Hospital and Medical Center, Phoenix, AZ 85013 USA

**Keywords:** *BRCA1/2*, Ovarian cancer, PARP, Platinum-refractory

## Abstract

**Background:**

Therapy for advanced epithelial ovarian cancer (OC) includes first line platinum/taxane-containing chemotherapy and re-treatment with platinum-containing regimens for disease recurrence in patients likely to respond again. Single-agent, non-platinum, cytotoxic agents are commonly used to treat patients resistant to platinum retreatment, but these agents are associated with dose-limiting toxicities and response rates below 20%.

**Main body:**

Recent advances have led to novel targeted treatments for recurrent OC that offer opportunities to improve response rates and prolong progression-free intervals. However, they also add complexity to the process of selecting treatment for individual patients at different stages of the disease process. Advanced and recurrent OC is rarely cured. Multiple lines of platinum combinations, and nonplatinum chemotherapeutics eventually fail to achieve clinical benefit, thus other active and tolerable systemic therapies are needed. Consequently, the US Food and Drug Administration has created a mechanism for “accelerated approval” of new medicines in situations of high unmet medical need.

**Conclusion:**

We review the clinical implications of recent key clinical studies in these settings and outline the path forward for study design and approval of novel therapeutics to treat recurrent OC.

## Introduction

More than 70% of women with epithelial ovarian cancer (OC), which typically also includes fallopian tube and primary peritoneal cancers, have advanced disease at the time of first diagnosis. Although many patients with advanced disease achieve complete remission after surgical cytoreduction and platinum- and taxane-based chemotherapy, up to 80% eventually experience recurrence [1]. Two major goals of recent and ongoing clinical studies in OC have been to achieve a more durable disease-free interval after induction therapy and better response rates for regimens administered beyond first line therapy. We provide a succinct overview of recent studies addressing these two goals and outline the unmet need for additional treatment options.

## Review

### What is the role of maintenance therapy as part of first line therapy?

Platinum-containing induction chemotherapy remains a standard first-line treatment for women with advanced OC. However, there has been vigorous debate regarding the role of maintenance chemotherapy in patients with advanced OC who achieve an objective response during induction chemotherapy [1, 2]. In the 1990s, studies of extended platinum chemotherapy (8–12 cycles) found no evidence for improved progression-free survival (PFS) or overall survival (OS) versus 5–6 cycles (reviewed in Markman 2015) [2]. Furthermore, extended platinum regimens were associated with increased toxicity versus standard regimens.

In the early 2000s, a true maintenance study assessed paclitaxel maintenance therapy in women who had achieved an objective complete response to induction platinum-paclitaxel [3]. Patients were randomized to either 12 or 3 additional cycles of single-agent paclitaxel. The study was terminated early because patients in the 12-cycle arm had significantly longer PFS than patients in the 3-cycle arm. At mature follow-up, PFS was 22 versus 14 months (*P* = 0.006), but there was no significant effect on OS (53 versus 48 months; *P* = 0.34) [4]. The lack of a significant effect on OS may have several explanations, including exposure to subsequent active treatment regimens [2]. Patients in the 12-cycle arm experienced higher rates of peripheral neuropathy. Another study of single-agent paclitaxel (6 cycles) after complete or pathologic response to platinum-paclitaxel induction demonstrated no improvement in PFS or OS, and increased rates of peripheral neuropathy in the paclitaxel arm [5]. Recently, those findings were confirmed by another phase 3 study (GOG-212), which also demonstrated no OS benefit for patients who received maintenance paclitaxel [6].

Two studies (GOG-218 and ICON7) have explored use of the anti-angiogenesis agent bevacizumab to extend the disease-free interval after first line chemotherapy [7, 8]. In both studies, bevacizumab was added to standard chemotherapy (5 or 6 cycles of carboplatin-paclitaxel), and bevacizumab monotherapy was continued (for 12–22 cycles) after cessation of chemotherapy. Initially, both studies reported improved PFS when extended bevacizumab treatment was added to chemotherapy [7, 8]. However, long-term follow-up of the ICON7 study found no significant improvement in PFS or OS in the overall study population, although there was evidence of benefit in high-risk patients [9]. The GOG-218 study had three treatment arms, all of which received 6 cycles of carboplatin-paclitaxel [7]. One arm received bevacizumab concurrent with chemotherapy, a second arm received bevacizumab concurrently and during an extended period (up to cycle 22), and the third arm received only chemotherapy. A placebo was administered as appropriate control in this double-blind study. Patients in the extended-bevacizumab arm had the longest PFS (14.1 months), which was significantly longer than the chemotherapy-alone arm (10.3 months; hazard ratio [HR]: 0.717; 95% confidence interval [CI]: 0.625–0.824; *P* < 0.001). The PFS in the concurrent-bevacizumab arm was 11.2 months [7]. In both studies, bevacizumab was associated with increased risk of adverse events, especially gastrointestinal events [10], and neither study found a benefit in terms of OS for the overall study population. Exposure to bevacizumab or other active regimens after the study may have confounded any OS effect; nonetheless, the role of bevacizumab in front-line therapy for advanced OC—either concurrently with chemotherapy or for an extended duration—continues to be controversial. SOLO-1 is an ongoing study of olaparib maintenance monotherapy after first line platinum-based chemotherapy. Also, the randomized, phase 3 PAOLA-1 study (NCT02477644) is comparing olaparib and bevacizumab versus placebo and bevacizumab as maintenance therapy in patients with advanced OC following first line treatment with platinum chemotherapy and bevacizumab [11]. Olaparib and other Poly (ADP-ribose) polymerase (PARP) inhibitors are discussed in more detail below.

### Recurrent ovarian cancer

**Fig. 1:**
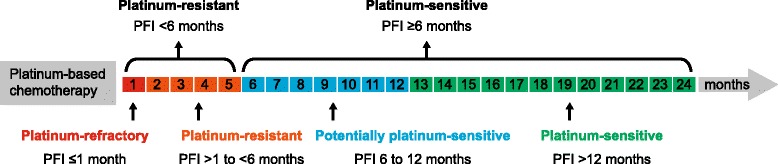
Definitions of Platinum-Refractory, Platinum-Resistant, Potentially Platinum-Sensitive, and Fully Platinum-Sensitive Ovarian Cancer. Patients with ovarian cancer are classified broadly in two main categories: “platinum-resistant” if the platinum-free interval (PFI) is less than 6 months, and “platinum-sensitive” if the PFI is at least 6 months. A more specific classification defines patients with ovarian cancer as “platinum-refractory” if disease progression occurs during chemotherapy or within 4 weeks after the last dose, “platinum-resistant” if the PFI is greater than 1 month and less than 6 months since last line of platinum-based therapy, “potentially platinum-sensitive” if the PFI is between 6 and 12 months, and “platinum-sensitive” if the PFI is more than 12 months

Selection of treatment for recurrence of advanced epithelial OC is generally guided by the progression-free interval [12] (Fig. 1). When the time to progression is >6 months after cessation of initial platinum-containing chemotherapy, the disease is considered to be platinum-sensitive. In these patients, treatment using a combination platinum-containing chemotherapy (typically including a taxane, pegylated liposomal doxorubicin [PLD], gemcitabine, or bevacizumab in carefully selected patients) is considered a preferred treatment option [13]. Response rates to second line, combination platinum-containing chemotherapy in patients with platinum-sensitive tumors are approximately 50%–65% [14–16]. Combination therapy demonstrated advantages over single-agent platinum regimens in terms of both PFS and OS [17]. When progression after first line therapy occurs less than 6 months after cessation of chemotherapy, the disease is considered to be platinum-resistant; recommended second line therapies in these patients include mostly single-agent, nonplatinum-containing chemotherapy regimens, with the possible addition of bevacizumab or pazopanib in carefully selected patients [13]. When progression occurs during chemotherapy or within 1 month of cessation, the disease is considered platinum-refractory [12].

**Fig. 2:**
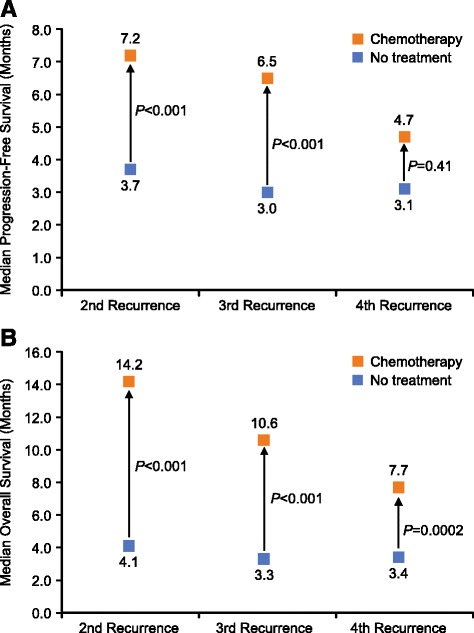
Median Progression-Free Survival (**a**) and Overall Survival (**b**) Associated with Successive Lines of Chemotherapy (Versus no Treatment) in a Retrospective Analysis of Three Randomized Trials in Patients with Advanced Ovarian Cancer [19]. Data from Hanker et al. *Ann Oncol*. 2012;23:2605–12. Hanker et al. performed a retrospective, pooled analysis of three randomized, phase 3 studies of primary taxane-platinum-based chemotherapy. The analysis included 1620 patients for whom complete data were available. Responsiveness to platinum-containing regimens declined dramatically after 2 prior *lines*, even in patients who were initially platinum-sensitive [19]

Until recent years, there were essentially no treatment options other than repeated courses of chemotherapy in patients with 2 or more prior lines of chemotherapy. Furthermore, nearly all patients eventually become resistant to platinum-containing regimens [12]. Thus, the concept of platinum sensitivity becomes less important beyond 2 or 3 lines of chemotherapy, and its relevance to treatment selection in such patients is not well understood. The limited available evidence indicates that responsiveness to platinum-containing regimens declines dramatically after 2 prior lines, even in patients who were initially platinum-sensitive. In one study of 63 patients who received at least 3 lines of chemotherapy, only 11.9% had a clinical response to third line chemotherapy, although 52% had responded to second line [18]. Nonplatinum-containing regimens—such as PLD, paclitaxel, gemcitabine, or topotecan—have similar response profiles (range 10%–15%), PFS (3–4 months), and OS (~12 months) when used as late-line therapies [12]. A retrospective analysis of 3 large European clinical studies of chemotherapy in patients with OC (*N* = 1620) found that the benefits of chemotherapy in terms of increased PFS or OS declined with successive recurrences (Fig. 2) [19]. Although chemotherapy for a fourth recurrence was still associated with a small benefit in terms of OS, the authors concluded that this benefit was mostly due to patients with platinum-sensitive disease, and that chemotherapy beyond three recurrences was not beneficial in patients with platinum-resistant disease. This conclusion, however, may need to be revisited as more data accumulate from studies of patient subgroups, and from new and emerging treatment strategies for patients with multiple recurrences.

Because of reduced potential for benefit after 2 prior lines of chemotherapy, and severe effects on quality of life (QOL), some patients forego chemotherapy after 2 prior lines, although many patients prefer to continue receiving additional lines of chemotherapy even if it confers diminishing benefits [20]. Undoubtedly, many patients withstand the side effects from subsequent lines of chemotherapy but obtain limited benefit. Furthermore, repeated chemotherapy regimens may expose patients to cumulative toxicities associated with many of these regimens [1, 19].

Recently, molecularly targeted inhibitors of vascular endothelial growth factor (VEGF; ie, bevacizumab) and PARP (ie, olaparib, rucaparib, and niraparib) have emerged as treatment options in patients with advanced epithelial OC after multiple prior lines of chemotherapy (Table 1) [21–32]. Both bevacizumab and PARP inhibitors require careful patient selection, the criteria for which are still evolving.

**Table 1:** Clinical activity of targeted therapies for treatment of recurrent ovarian cancer in heavily pretreated patients

	Phase	Patients with OC, n	Previous therapies	ORR, n (%)	CR, n (%)	Median DoR, mo	Median PFS, mo	OS, mo
Bevacizumab Monotherapy								
Cannistra 2007 [21]	2	44	2–3	7 (16)	0	4.2	4.4	10.7
Burger 2007 [22]	2	62	1–2	13 (21)	2 (3)	10.3	4.7	16.9
Monk 2006 [23]		32	5 (range: 2–10)	5 (16)	1 (3)	NR	5.5	6.9
Pietzner 2011 [24]		15	5.4 (range: 1–7)	2 (13)	0	NR	NR	15.0
Bevacizumab-Chemotherapy Combination								
AURELIA [25]	3	361	≤2	27.3%	NR	NR	6.7	16.6
OCEANS [26, 27]	2	484	≤1	190/242 (78.5)	42 (17)	10.4	12.4	33.6
GOG-213 [28]	3	674	≥3	196/249 (78)	79/249 (32)	NR	13.8	42.2
Olaparib Monotherapy								
Kaufman 2015 [29]	2	193	4.3 ± 2.2 (SD)	60 (31)	6 (3)	7.5	7	16.6
≥ 3 Prior lines [30]^a^		137^a^	≥3	46 (34)	2 (2)	7.9	6.7	NR
Gelmon 2011 [31]	2	65	3 (range: 1–10)	18 (29)	0	NR	7.3	NR
Rucaparib Monotherapy								
Swisher 2017 [32]	2							
*BRCA* mutant		40	1–2	32 (80)	NR	9.2	12.8	NR
*BRCA* wild-type								
LOH high		82	1–2	24 (29)	NR	10.8	5.7	NR
LOH low		70	1–2	7 (10)		5.6	5.2	

*CR* complete response, *DoR* duration of response, *LOH* loss-of-heterozygosity score, *mo* months, *NR* not reported, *OC* ovarian cancer, *ORR* objective/overall response rate, *OS* overall survival, *PFS* progression-free survival, *SD* standard deviation

^a^Subset of patients in Kaufman/Domchek who had measurable disease at baseline and ≥3 prior lines of chemotherapy

### Which patients are candidates for bevacizumab in recurrent OC

Bevacizumab, in combination with paclitaxel, PLD, or topotecan is approved for the treatment of patients with platinum-resistant recurrent epithelial OC who received no more than 2 prior lines of chemotherapy [21, 22, 25, 33]. In the AURELIA study, patients with platinum-resistant OC and 2 or fewer prior lines of chemotherapy had response rates of 27.3% in the bevacizumab plus chemotherapy arm versus 11.8% in the chemotherapy-alone arm (*P* = 0.001). Note that this study excluded patients who were platinum-refractory (progression during previous platinum-containing therapy) [25].

In patients with platinum-sensitive, recurrent OC, the OCEANS study found significantly increased objective response rate (ORR; 78% versus 57%; *P* < 0.001) and PFS (12.4 versus 8.4 months; *P* < 0.001) when bevacizumab was added to gemcitabine plus carboplatin, but there was no improvement in OS [26, 27]. Another randomized phase 3 study (GOG-213) found a statistically significant improvement in response rate (78% versus 59% in a subset of patients with evaluable data from imaging; *P* < 0.001) and PFS (13.8 versus 10.4 months; *P* < 0.001) when bevacizumab was added to paclitaxel plus carboplatin [28]. In the primary analysis of this study, OS was better in the group that received bevacizumab (42.2 versus 37.7 months), although this incremental improvement narrowly missed achieving statistical significance (*P* = 0.056). However, when miscalculations of the prior platinum-free interval were corrected, the difference in OS achieved statistical significance (*P* = 0.045) [28].

Single-agent bevacizumab has also shown activity (clinical response rate 16%–21%) in patients with 1 to 3 prior lines of chemotherapy, most of whom were platinum-resistant [21, 22]. Other small studies have reported responses to single-agent bevacizumab as a later line of therapy in patients with recurrent, platinum-resistant OC, with response rates ranging from 13% to 16% [21, 23, 24].

Thus, there is evidence to support the use of bevacizumab in multiple settings, including in combination with chemotherapy (typically PLD, weekly paclitaxel, or topotecan) in platinum-resistant patients with no more than 2 prior lines of chemotherapy. But bevacizumab may also provide benefit in platinum-sensitive patients in combination with carboplatin-gemcitabine or carboplatin-paclitaxel. The United States Food and Drug Administration (US FDA)-approved indications for bevacizumab in OC are summarized in Table 2 [33]. Perhaps just as important to the selection of patients for bevacizumab is the strict exclusion of patients who are at increased risk of bowel perforation. Restrictive exclusion criteria used in clinical studies are commonly followed [21, 25]. These criteria exclude any patient with a history of bowel obstruction (including subocclusive disease) related to underlying disease, history of abdominal fistula, gastrointestinal perforation, intra-abdominal abscess, evidence of recto-sigmoidal involvement by pelvic exam, bowel involvement on computed tomography, or clinical symptoms of bowel obstruction [25]. The role of bevacizumab after previous exposure requires further study; thus, some clinicians will withhold repeat courses until further data are reported.

**Table 2:** US FDA-approved targeted therapies for ovarian cancer

	Drug class	Ovarian cancer indication	Black box warnings	Warnings and precautions
Bevacizumab [33]	VEGF inhibitor; anti-angiogenesis	Platinum-resistant recurrent disease • In combination with paclitaxel, PLD, or topotecan with no more than 2 prior lines of chemotherapy Platinum-sensitive recurrent disease • In combination with carboplatin and paclitaxel, or carboplatin and gemcitabine; followed by single-agent bevacizumab	• Gastrointestinal perforations • Surgery and wound healing complications • Hemorrhage	• Perforation or fistula • Arterial and venous thromboembolic events • Hypertension • Posterior reversible encephalopathy syndrome • Proteinuria • Infusion reactions • Embryo-fetal toxicity • Ovarian failure
Niraparib [36]	PARP inhibitor	Maintenance treatment of recurrent disease in complete or partial response to platinum-based chemotherapy	None	• Myelodysplastic syndrome/acute myeloid leukemia • Bone marrow suppression • Cardiovascular effects (blood pressure and heart rate) • Embryo-fetal toxicity
Olaparib [35]	PARP inhibitor	Maintenance treatment of recurrent disease in complete or partial response to platinum-based chemotherapy Treatment of deleterious or suspected deleterious germline *BRCA*-mutated disease with ≥3 prior lines of chemotherapy; requires FDA-approved companion diagnostic test	None	• Myelodysplastic syndrome/acute myeloid leukemia • Pneumonitis • Embryo-fetal toxicity
Rucaparib [34]	PARP inhibitor	Monotherapy in patients with deleterious *BRCA* mutations treated with two or more prior chemotherapies; requires companion diagnostic test	None	• Myelodysplastic syndrome/acute myeloid leukemia • Embryo-fetal toxicity

*PARP* poly (ADP-ribose) polymerase, *PLD* pegylated liposomal doxorubicin, *VEGF* vascular endothelial growth factor

### What is the role of PARP inhibitors for treatment of recurrent OC?

Recent clinical studies have evaluated the potential roles of PARP inhibitors for treatment of patients with advanced OC in two distinct settings: 1) when disease has recurred or progressed after 2–3 or more prior lines of platinum-containing chemotherapy, and 2) when the disease is in a state of response after completion of a recent course of platinum-containing chemotherapy (maintenance therapy). Some of these studies enrolled only patients with known deleterious *BRCA* mutations (germline or somatic).

### Which patients with recurrent ovarian cancer are candidates for a PARP inhibitor?

In late 2014, olaparib received accelerated approval by the US FDA as monotherapy for patients with advanced OC harboring deleterious or suspected deleterious germline *BRCA* (g*BRCA*) mutations and who were previously treated with 3 or more lines of chemotherapy. Approval was primarily based on data from a single-arm study of patients with advanced OC and g*BRCA1/2* mutations [29, 30], most of whom had received multiple prior lines of chemotherapy (mean 4.3). Among 137 patients who had measurable disease at baseline and who had received 3 or more prior lines of chemotherapy, the ORR was 34% and the median duration of response was 7.9 months (Table 1) [30]. Although patients with platinum-sensitive disease had the highest ORR (18/39; 46%), the response rate observed in patients with platinum-resistant disease (24/81; 30%) suggests that platinum resistance does not preclude responsiveness to olaparib as late-line therapy in patients with OC and a *BRCA* mutation. In contrast, the ORR was 14% (2/14) among patients with platinum-refractory disease [30].

In late 2016, another PARP inhibitor, rucaparib (Table 2), was approved for treatment of patients with advanced OC associated with deleterious *BRCA* mutation (germline or somatic) and who had progressed after 2 or more prior lines of chemotherapy. The accelerated approval was based upon ORR (54%) [34] and median duration of response (9.2 months) in patients with a *BRCA* mutation from the single-arm, phase 2 study (ARIEL2 Part 1) that enrolled women with high-grade, relapsed, platinum-sensitive OC [32]. The ARIEL2 study results are shown in Table 1 [32].

One of the goals of the ARIEL2 study was to explore biomarkers of response to PARP inhibition. Thus, the study also included patients who had wild-type *BRCA*, but tumor samples were analyzed for genetic loss of heterozygosity (LOH) as a potential surrogate marker of homologous recombination deficiency (HRD). Although patients with wild-type *BRCA* but high LOH scores had lower rates of ORR and shorter PFS than patients with *BRCA* mutation, both cohorts (those with *BRCA* mutation and those with wild-type *BRCA* but high LOH scores, indicating presence of HRD) had ORR and PFS that were significantly better than patients with wild-type *BRCA* and low LOH scores (*P* < 0.02) [32]. These results demonstrated that PARP inhibitors appear active in a broader set of patients than only those harboring deleterious *BRCA* mutations. Part 2 of the ARIEL2 trial is ongoing, and it will prospectively evaluate rucaparib responsiveness in patient subgroups defined by LOH scores.

PARP inhibitors are also associated with side effects, although not usually as severe as those observed with chemotherapy. However, a small percentage (1% or less) in both olaparib [35] and rucaparib [34] studies developed myelodysplastic syndrome/acute myeloid leukemia. Patients should be monitored for hematologic toxicities at baseline and during treatment.

### Is there a role for PARP inhibitors in maintenance therapy?

Both olaparib [35] and niraparib [36] (Table 2), have been approved as maintenance therapies in patients who are in a complete or partial response to platinum-based chemotherapy [37–39]. Some have questioned use of the term “maintenance therapy” in this setting, on the basis that many patients had only a partial response to chemotherapy rather than a complete response. Nevertheless, this term has been adopted by regulatory agencies, and it will continue to be used in this context.

The maintenance therapy studies of PARP inhibitors enrolled patients with OC who were in response (partial or complete) after their most recent platinum-containing regimen and who had responded for at least 6 months after the preceding platinum-containing regimen. In the phase 2 olaparib study (Study 19), patients in the olaparib arm had significantly longer PFS than those in the placebo arm (8.4 versus 4.8 months; *P* < 0.001) [37]. Further analysis according to *BRCA* status revealed that patients with a deleterious *BRCA* mutation (germline or somatic) had the greatest benefit from olaparib versus placebo (PFS 11.2 versus 4.3 months; *P* < 0.001), but even patients with wild-type *BRCA* benefited from olaparib maintenance therapy (PFS 7.4 versus 5.5 months; *P* = 0.007) [40]. While no significant difference in OS was found for the general population, patients with *BRCA* mutation-positive, platinum-sensitive, recurrent OC benefited from longer survival when treated with olaparib [41]. The recently reported SOLO-2 study was a confirmatory phase 3 trial to determine the efficacy of olaparib tablets as maintenance monotherapy in patients with platinum-sensitive, relapsed OC and germline *BRCA*-mutation [39]. In this study, olaparib maintenance therapy was associated with marked improvements in PFS versus placebo (Table 3). Health-related QOL also was evaluated in the SOLO-2 study. Patients maintained their QOL while on olaparib maintenance therapy, exhibiting no significant negative effect on health-related QOL versus placebo. Patients receiving olaparib (versus placebo) experienced a significant improvement in multiple assessments of patient-centered benefits [42].

**Table 3:** Phase 3 studies of PARP inhibitors for maintenance therapy in patients with platinum-sensitive ovarian cancer

	Prior lines of chemotherapy	Inclusion biomarkers	Median PFS, months		HR for PFS (95% CI)	*P* value
			Active therapy	Placebo		
Niraparib Monotherapy						
NOVA [38]	≥2	None Patients stratified according to *gBRCA* status and HRD score	*gBRCA*: 21.0	5.5	0.27 (0.17–0.41)	<0.001
			Non-*gBRCA*: 9.3	3.9	0.45 (0.34–0.61)	<0.001
			HRD-positive: 12.9	3.8	0.38 (0.24–0.59)	<0.001
			HRD-negative: 6.9	3.8	0.58 (0.36–0.92)	0.02
Olaparib Monotherapy						
SOLO-2 [39]	≥2	*BRCA1/2* mutation	30.2	5.5	0.25 (0.18–0.35)	<0.001
Rucaparib Monotherapy						
ARIEL3 [45, 46]	≥3^a^	None	*BRCA* mutation: 16.6 HRD-positive: 13.6 ITT population: 10.8	5.4 5.4 5.4	0.23 0.32 0.36	<0.001 <0.001 <0.001

*CI* confidence interval, *gBRCA* germline *BRCA* mutation, *HR* hazard ratio, *HRD* homologous recombination deficiency, *ITT* intent-to treat, *PARP* poly (ADP-ribose) polymerase, *PFS* progression-free survival

^a^Received ≥2 prior platinum-based treatment regimens including platinum based regimen and no more than 1 non-platinum chemotherapy regimen

The phase 3 niraparib study (NOVA) enrolled 2 cohorts of patients according to the presence or absence of a g*BRCA* mutation [38]. In the g*BRCA* cohort, niraparib treatment was also associated with markedly longer PFS than placebo (Table 3). Patients in the non-g*BRCA* cohort also had significant benefits from niraparib therapy. Interestingly, even patients who did not have a g*BRCA* mutation, and who did not exhibit HRD, experienced a longer PFS with niraparib versus placebo [38]. In a subgroup analysis, niraparib provided significant benefit in patients with recurrent OC who achieved a partial response following platinum therapy [43]. In addition, 49% of the total patient population was found to have developed platinum resistance to previous chemotherapy, yet the study met its primary endpoint of prolonged PFS following response to most recent platinum therapy [44]. Results for OS have not yet been reported from either the SOLO-2 or NOVA studies.

With appropriate caution regarding toxicities, PARP inhibitors are emerging as a potential maintenance therapy in patients with OC who have responded to at least 2 prior lines of platinum-containing chemotherapy and are in response (complete or partial) to the most recent course. Although *BRCA* mutations and deficiency in homologous recombination repair appear to be relative markers predictive of response to PARP inhibitors, lack of these markers does not preclude a response. This concept is especially notable in the NOVA trial, which led to recent approval of niraparib irrespective of biomarker status. Thus, neither *BRCA* status nor HRD score are requisites for use of niraparib. The ongoing ARIEL3 study (NCT01968213) is exploring the use of rucaparib as maintenance therapy, with enrollment criteria similar to those in the olaparib and niraparib maintenance therapy studies, but without restrictions related to *BRCA* status. As reported in June 2017, the phase 3 ARIEL3 study demonstrated improved PFS (Table 3) by investigator review for rucaparib compared with placebo in all three primary efficacy analyses: *BRCA* mutation (16.6 months vs 5.4 months; HR: 0.23, *P* < 0.001); HRD-positive (13.6 months vs 5.4 months; HR: 0.32, *P* < 0.001); overall intent-to-treat populations (10.8 months vs 5.4 months; HR: 0.36, *P* < 0.001) [45, 46].

### What percentage of patients with advanced ovarian cancer have *BRCA* mutations?

In population-based studies of unselected patients with OC, 5%–18% of cases were found to be associated with g*BRCA* mutations [47]. Limited available data suggest that another 5%–10% arise from somatic *BRCA* mutations [48]. Thus, at initial diagnosis the percentage of patients with OC whose cancer is *BRCA*-related is modest. However, patients with *BRCA*-related OC have better long-term survival than non-carriers [49, 50], which may in part be related to better responsiveness to platinum-based chemotherapy [50, 51]. Thus, patient groups who have undergone multiple lines of chemotherapy may become enriched for *BRCA* mutation carriers.

### What are the prospects for the unmet needs of patients requiring third line therapy and beyond?

Several studies are investigating treatment options for patients who have platinum-resistant disease or who have progressed after multiple lines of treatment, including the third line setting and beyond (Table 4). With regard to PARP inhibitors, the phase 3 SOLO-3 study is measuring PFS for olaparib versus single-agent investigator’s choice nonplatinum-based chemotherapy in patients with platinum-sensitive high-grade serous OC or high-grade endometrioid cancer who progressed at least 6 months after last platinum treatment, and have received 2 or more platinum-based lines of therapy (NCT02282020) [52]. To determine the most sensitive and specific assays to assess HRD and more accurately predict patients who may respond to treatment with olaparib, the phase 2 LIGHT study (NCT02983799) is evaluating the efficacy and safety of olaparib in patients with platinum-sensitive, relapsed OC who have received ≥2 prior lines of platinum-based chemotherapy. Patients will be stratified by use of different HRD genetic tests [53].

**Table 4:** Ongoing phase 2 and 3 studies investigating late-line therapies in ovarian cancer

Agent	NCT # Study name	Phase	Est. N	Setting	Expected completion
PARP Inhibitors					
Niraparib	NCT02354586 QUADRA	2	400	Recurrent, ≥4th- 5th-line	October 2017
Olaparib	NCT02282020 SOLO-3	3	411	Recurrent, ≥3rd-line	December 2017
Olaparib	NCT02889900 CONCERTO	2	100	Recurrent, ≥3rd-line	November 2018
Rucaparib	NCT01891344 ARIEL2	2	480	Recurrent, ≥4th-line	March 2017
Rucaparib	NCT01968213 ARIEL3	3	540	Recurrent, ≥3rd-line	March 2017
Mirvetuximab soravtansine (an antibody-drug conjugate targeting the folate-alpha receptor)					
	NCT02631876 FORWARDI	3	333	Platinum-resistant; 1–3 prior lines of chemotherapy	February 2019
NUC-1031 (gemcitabine prodrug^a^)					
	NCT03146663	2	64	Platinum-resistant; ≥3 prior lines of chemotherapy	June 2020
Trabectedin (novel alkylating chemotherapy agent)					
	NCT01846611 ORCHYD	3	670	Platinum-sensitive; 3rd line; known *BRCA1/2* mutation	December 2019
Ipilimumab (immune checkpoint inhibitor)					
	NCT01611558	2	49	Platinum-sensitive; ≤4 prior lines of chemotherapy	July 2019
Birinapant (SMAC mimetic and IAP inhibitor)					
	NCT02756130	2	34	In combination with carboplatin in newly diagnosed or recurrent disease	June 2020
Volasertib (Plk1 inhibitor)					
				In development; no ongoing phase 2 or phase 3 studies	

*IAP* inhibitor of apoptosis protein, *Plk1* polo-like kinase 1, *SMAC* second mitochondrial-derived activator of caspases

^a^Prodrug is a compound that is metabolized into a pharmacologically active drug after administration

The combination of olaparib with cediranib, an antiangiogenic VEGF receptor inhibitor, is of interest. The phase 2 NCI-2012-02938 study (NCT01116648) in women with recurrent platinum-sensitive OC reported significantly longer median PFS for those treated with combination therapy versus olaparib alone (16.5 vs 8.2 months, HR: 0.50, *P* = 0.007). This effect was greatest for patients without known g*BRCA* mutations: median PFS was 23.7 versus 5.7 months (HR: 0.32, *P* = 0.002) and median OS was 37.8 versus 23.0 months (HR: 0.48, *P* = 0.074) with combination therapy versus olaparib alone, respectively. These results suggest that the combination of a PARP inhibitor and an antiangiogenic may result in increased activity in these patients [54]. Ongoing studies for this combination include: the phase 3 NRG-GY004 study (NCT02446600) in platinum-sensitive OC compared to olaparib alone or platinum-based chemotherapy [55]; the single arm phase 2 CONCERTO study (NCT02889900) in women with platinum-resistant relapsed disease without a g*BRCA* mutation [56]; and the phase 2/3 NRG-GY005 study (NCT02502266) in patients with platinum-resistant disease who have received no more than 3 prior treatment regimens [57].

The phase 2 QUADRA study (NCT02354586) is evaluating the antitumor activity of niraparib in patients with advanced, relapsed, high-grade serous epithelial ovarian, fallopian tube, or primary peritoneal cancer who received 3 or more prior chemotherapy regimens [58]. In platinum-resistant disease, ongoing studies are investigating the use of the antibody-drug conjugate mirvetuximab soravtansine and the gemcitabine prodrug NUC-103. Other agents are also being investigated in phase 2 studies, including the alkylating agent trabectedin, an antibody-drug conjugate targeting the folic acid receptor (mirvetuximab soravtensine), the immune checkpoint inhibitors ipilimumab, durvalumab, and tremelimumab, and the targeted agents birinapant and volasertib (Table 4). AZD1775, an inhibitor of the WEE1 tyrosine kinase, is also is being explored in platinum-resistant OC (NCT02272790) [59]. Numerous other agents are being studied as earlier lines of therapy for advanced OC, including the folic acid receptor antibody farletuzumab and several inhibitors of histone deactylase (HDAC). The HDAC inhibitors most clinically advanced for treatment of OC are entinostat (NCT02915523), vorinostat (NCT00132067), and ricolinostat (NCT02661815). Finally, several vaccines are being studied as potential treatments for advanced OC [60, 61]. Selected examples include dendritic cell vaccines, patient-specific autologous tumor cell vaccines, and vaccines targeting various antigens enriched in tumor cells, such as folate receptor alpha, HER2, brachyury, insulin-like growth factor binding protein-2, survivin, and carcinoembryonic antigen. These emerging therapies are of interest owing to the very limited treatment options for women who have failed 2 or more lines of chemotherapy, including platinum-based agents, and who have received or were ineligible to receive bevacizumab or a PARP inhibitor.

Another important aspect of addressing the unmet need for treatments in advanced OC is the regulatory pathway for accelerated approval in the United States [62, 63]. The Accelerated Approval Program allows earlier approval of drugs that treat serious conditions and fill an unmet need. In this program, oncology drugs can be approved based on a surrogate endpoint (such as objective response rate rather than survival), when those drugs fill an unmet need, have acceptable toxicity, and satisfy criteria for chemistry, manufacturing, and controls. Typically, approval is provisional and a confirmatory phase 3 trial is expected to be undertaken. This program has been beneficial for patients with OC, which has lagged behind other cancers with regard to treatment options.

A good example of how accelerated approval has aided the OC community is PLD, which was granted accelerated approval in 1999. Under accelerated approval, which was based on three phase 2 studies, PLD was indicated for the treatment of metastatic OC in patients with disease that was refractory to both paclitaxel- and platinum-based chemotherapy. According to the terms of the accelerated approval, a randomized, phase 3 clinical study was then completed to formally demonstrate the drug’s clinical benefit in patients with relapsed OC [64, 65]. On the basis of that trial, full approval of PLD was granted in 2005. More recently, the PARP inhibitor olaparib was granted accelerated approval in 2014 and the phase 3 SOLO-2 trial was submitted as a confirmatory study; the ongoing SOLO-3 trial may serve as a second confirmatory study. Rucaparib was also granted accelerated approval in 2016, with ARIEL3 and ARIEL4 serving as confirmatory studies. Given the continuing unmet need for therapies in many patients with OC, it is encouraging that the accelerated approval program exists to usher in new treatment options in a manner that allows access that ensures an appropriate level of patient safety.

Despite new therapeutic strategies approved in recent years and promising strategies and agents on the horizon, there continue to be unmet needs for patients with advanced OC. Addressing those needs will require a reexamination and possibly a redesign of the drug discovery and development process. The cancer drug development process is facing many challenges, including inefficient clinical study designs, relative paucity of new drug targets but a proliferation of “*me-too*” drugs, and the dilution of the patient population available for enrollment into clinical studies. That dilution has many contributing causes, including the progress toward personalized medicine in which few patients may qualify for a given treatment, as well as the proliferation of clinical studies needed to test the large numbers of drugs with similar or identical mechanisms of action. Novel study designs including master protocols for umbrella, basket, and platform studies are being used to address this need [66]. Furthermore, many emerging therapies require biomarker tests, which must be developed and approved, and which are often expensive and provide low yields. These problems extend well beyond the OC arena, and their solutions call for a concerted and creative effort on the part of the scientific, pharmaceutical, and regulatory communities.

## Conclusions

Platinum-based chemotherapy is recommended as first line therapy in women with advanced epithelial OC. Bevacizumab may have roles in selected patients with recurrent disease in combination with 5 approved chemotherapy backbones. For those patients who achieve an objective response to retreatment with platinum-based chemotherapy, a recent phase 3 study showed that niraparib can extend PFS when used as maintenance monotherapy. The recently reported phase 3 SOLO-2 study confirmed the efficacy of olaparib tablets as maintenance monotherapy in patients with platinum-sensitive, relapsed OC with a g*BRCA* mutation. Currently, there are limited treatment options for women with recurrent OC who have failed two or more lines of chemotherapy and have received or were ineligible to receive bevacizumab or a PARP inhibitor [39]. The US FDA’s accelerated approval of olaparib and rucaparib for the treatment of recurrent disease offers exciting new treatment options. Accelerated FDA approval poses an opportunity for additional new medicines to become rapidly available to address unmet needs.

## Abbreviations

CI: Confidence interval
CR: Complete response
DoR: Duration of response
g*BRCA*: germline *BRCA*
HR: Hazard ratio
HRD: Homologous recombination deficiency
ITT: Intent-to-treat
LOH: Loss of heterozygosity
NR: Not reported
OC: Ovarian cancer
ORR: Objective response rate
OS: Overall survival
PARP: Poly(ADP-ribose) polymerase
PFI: Platinum-free interval
PFS: Progression-free survival
PLD: Pegylated liposomal doxorubicin
QOL: Quality of life
SD: Standard deviation
VEGF: Vascular endothelial growth factor
